# Microbial transformation of artemisinin by *Aspergillus terreus*

**DOI:** 10.1186/s40643-017-0164-6

**Published:** 2017-07-17

**Authors:** Hongchang Yu, Baowu Zhu, Yulian Zhan

**Affiliations:** 1 School of Life and Environmental Sciences, Guilin University of Electronic Technology, Guilin, 541004 People’s Republic of China; 2 State Key Laboratory of Bioreactor Engineering, East China University of Science and Technology, Shanghai, 200237 People’s Republic of China

**Keywords:** Microbial transformation, Artemisinin, *Aspergillus terreus*

## Abstract

**Background:**

Artemisinin (**1**) and its derivatives are now being widely used as antimalarial drugs, and they also exhibited good antitumor activities. So there has been much interest in the structural modification of artemisinin and its derivatives because of their effective bioactivities. The microbial transformation is a promising route to obtain artemisinin derivatives. The present study focuses on the microbial transformation of artemisinin by *Aspergillus terreus*.

**Results:**

During 6 days at 28 °C and 180 rpm, *Aspergillus terreus* transformed artemisinin to two products. They were identified as 1-deoxyartemisinin (**2**) and 4α-hydroxy-1-deoxyartemisinin (**3**) on the basis of their spectroscopic data.

**Conclusions:**

The microbial transformation of artemisinin by *Aspergillus terreus* was investigated, and two products (1-deoxyartemisinin and 4α-hydroxy-1-deoxyartemisinin) were obtained. This study is the first to report on the microbial transformation of artemisinin by *Aspergillus terreus*.

## Background

**Fig. 1:**
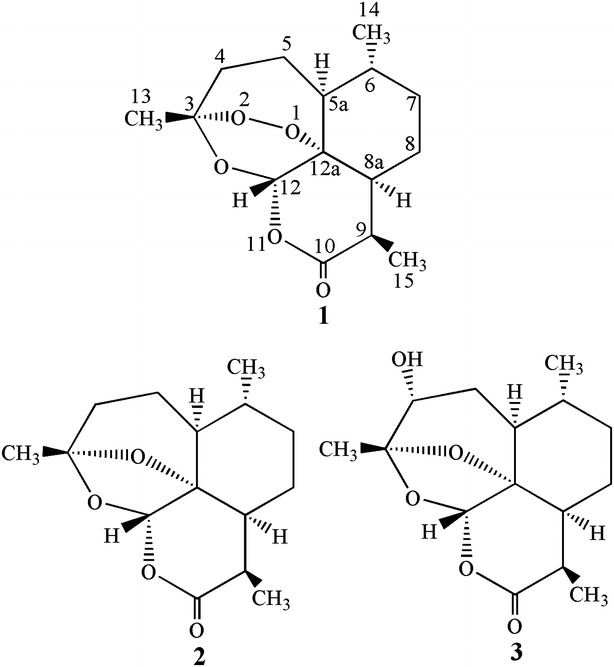
Structures of artemisinin and two products from microbial transformation of artemisinin by *Aspergillus terreus*

Artemisinin (Fig. 1) **1** (qinghaosu) is a sesquiterpene lactone and its structure was determined by X-ray analysis (Liu et al. 1979). Artemisinin and its derivatives such as dihydroartemisinin, artemether, artesunate, and arteether are now being widely used as antimalaria drugs. In some reports, artemisinin derivatives also exhibited good antitumor activities (Wu et al. 2004; Efferth et al. 2001, 2004; Singh and Lai 2001). There has been much interest in the structural modification of artemisinin and its derivatives because of their effective bioactivities. In this study, we report the microbial transformation of **1** by *Aspergillus terreus*, and two products were obtained.

## Methods

### General

^1^H NMR (nuclear magnetic resonance) and ^13^C NMR spectra were recorded in CDCl_3_ (chloroform-d) on a Bruker AV 500 MHz spectrometer. Chemical shifts were reported in ppm (*δ*), and *J* values were reported in Hz.

### Microorganism

*Aspergillus terreus* strain ZYL050009 was isolated from soil samples collected from the yew planting base at Guilin, China. The isolate was identified by amplification of the nuclear ribosomal internal transcribed spacer (ITS) region, using the primers ITS1 (5′-TCC GTA GGT GAA CCT GCG G-3′) and ITS 4 (5′-TCC TCC GCT TAT TGA TAT GC-3′) (White et al. 1989). The amplicons were sequenced, and alignments were performed using BLASTN algorithm. Reference sequences with the highest identity were selected and imported into the open source software MEGA 7.0 (Kumar et al. 2016). For the phylogenetic analysis, tree constructions were done with the software MEGA 7.0 using the neighbor-joining method (Hall 2013). Bootstrap analysis was done using 1000-times resampled data.

### Medium

All culture and microbial transformation experiments were performed in the following medium: potato infusion is made by boiling 200 g of sliced potatoes in 1 L deionized water for 30 min and then filtering the broth through cheesecloth. Deionized water is added such that the total volume of the suspension is 1 L. 20 g dextrose is then added and the medium is sterilized by autoclaving at 121 °C for 30 min.

### Microbial transformation of artemisinin (**1**) by *Aspergillus terreus*

Well-developed fungal mycelia were transferred into 250-mL Erlenmeyer flasks containing 60 mL of medium from the surface of agar slants. Cultures were grown for 48 h on a rotary shaker at 28 °C with shaking at 180 rpm, and used to inoculate 51 250-mL shake flasks that contained 60 mL of medium. The cultures were then incubated for 48 h using the same conditions as before. Artemisinin (Mediplantex, Vietnam) was dissolved in acetone (25 mg/mL), filter-sterilized, and 0.4 mL of this solution was added to each flask. A total of 510 mg of artemisinin was transformed. The cultures were incubated for additional 6 days at 28 °C while shaking at 180 rpm. The mycelia were separated by filtration and discarded. The filtrate was extracted three times with an equal volume of ethyl acetate (EtOAc). The extract was evaporated to dryness under vacuum to afford a residue.

### Chromatographic conditions

A total of 1.01 g of residue was obtained from the broth. The residue was purified by silica gel column chromatography, using a petroleum ether (60–90 °C)–acetone mobile phase in a gradient mode, eluting with 10–30% acetone.

## Results and discussion

**Fig. 2:**
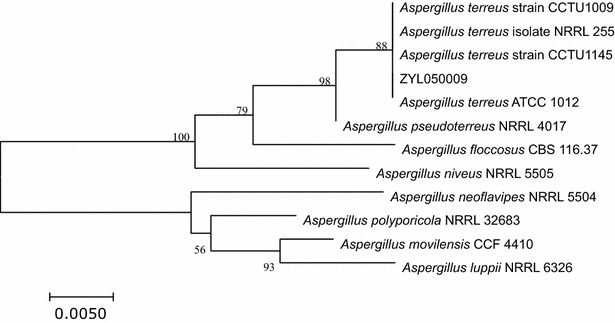
Phylogenetic tree based on the ITS sequences was generated using the neighbor-joining method and the MEGA7.0 software. All sequences data were retrieved from the GenBank database. Bootstrap values, expressed as percentages of 1000 replications, are given at branching points

We performed DNA sequencing for species identification. We isolated genomic DNA sample from the strain and sequenced the amplified ITS regions. A BLAST search of ITS rDNA sequences available in the GenBank database showed that 583-bp ITS from the strain shared 99% match with *A. terreus* ATCC 1012 (NR_131276.1), *A. terreus* strain CCTU1145 (GenBank: KY053120.1), *A. terreus* isolate NRRL 255 (GenBank: EF669586.1), and *A. terreus* strain CCTU1009 (GenBank: KY053112.1) (Fig. 2). The isolate was determined as *A. terreus*.

The microbial transformation of artemisinin by *A. terreus* resulted in 20 mg of **2** (yield 3.9%), 39 mg of **3** (yield 7.6%).

The structures of products were identified on the basis of their spectroscopic data. Data of ^1^H and ^13^C NMR spectra of product **2** were in agreement with the reported literatures’ data (Lee et al. 1989; Gaur et al. 2014). So product **2** was identified as 1-deoxyartemisinin (Fig. 1). The comparison of the ^1^H and ^13^C NMR data of product **3** with those of 4α-hydroxy-1-deoxyartemisinin (Parshikov et al. 2004; Zhan et al. 2015) was in complete agreement. Therefore, product **3** was confirmed to be 4α-hydroxy-1-deoxyartemisinin (Fig. 1).

### 1-deoxyartemisinin (**2**)

Colorless needles (from acetone); ^1^H-NMR (CDCl_3_, 500 MHz) *δ* 0.94 (3H, d, *J* = 5.6 Hz, Me-14), 0.99 (1H, m, H-8), 1.08 (1H, m, H-7), 1.19 (3H, d, *J* = 7.2 Hz, Me-15), 1.25 (3H, m, H-5, H-6, H-5a), 1.52 (3H, s, Me-13), 1.63 (1H, m, H-4), 1.78 (2H, m, H-4, H-7), 1.90 (2H, m, H-5, H-8), 2.00 (1H, m, H-8a), 3.18 (1H, m, H-9), 5.69 (1H, s, H-12); ^13^C NMR (CDCl_3_, 125 MHz) *δ* 171.8 (s, C-10), 109.2 (s, C-3), 99.6 (d, C-12), 82.4 (s, C-12a), 44.6 (d, C-5a), 42.4 (d, C-8a), 35.4 (d, C-6), 34.0 (t, C-4), 33.5 (t, C-7), 32.8 (d, C-9), 23.9 (q, C-13), 23.5 (t, C-8), 22.0 (t, C-5), 18.6 (q, C-14), 12.6 (q, C-15).

### 4α-hydroxy-1-deoxyartemisinin (**3**)

Colorless needles (from acetone); ^1^H-NMR (CDCl_3_, 500 MHz) *δ* 5.64 (1H, s, H-12), 3.62 (1H, brs, H-4β), 3.20 (1H, m, H-9), 2.06 (1H, m, H-8a), 1.99 (1H, m, H-5α), 1.93 (1H, m, H-8α), 1.82 (1H, m, H-7α), 1.58 (3H, s, Me-13), 1.54 (1H, m, H-5a), 1.50 (1H, m, H-5β), 1.29 (1H, m, H-6), 1.21 (3H, d, *J* = 7.2 Hz, Me-15), 1.13 (1H, m, H-7β), 1.00 (1H, m, H-8β), 0.93 (3H, d, *J* = 6.4 Hz, Me-14); ^13^C NMR (CDCl_3_, 125 MHz) *δ* 171.3 (s, C-10), 108.9 (s, C-3), 98.9 (d, C-12), 83.0 (s, C-12a), 69.1 (d, C-4), 42.1 (d, C-8a), 40.6 (d, C-5a), 35.1 (d, C-6), 33.4 (t, C-7), 32.7 (d, C-9), 30.3 (t, C-5), 23.5 (t, C-8), 20.5 (q, C-13), 18.4 (q, C-14), 12.6 (q, C-15).

Although artemisinin is effective against chloroquine-resistant parasites, its toxicities (Kamchonwongpaisan et al. 1997) and low solubility (Lin et al. 1989) in water limit its therapeutical use. The modification of artemisinin has been studied by chemical and biological methods in some reports (Gaur et al. 2014; Parshikov et al. 2004, 2006; Zhan et al. 2002; Goswami et al. 2010; Acton 1999). However, synthesis and semisynthesis of artemisinin derivatives are impossible or impracticable because of the complexity of the artemisinin molecule and the chemical lability of the peroxy ring system. Therefore, microbial transformation is a promising route to obtain artemisinin derivatives. There are many reports on microbial transformation of artemisinin by various microorganisms, such as *Aspergillus niger*, *Rhizopus stolonifer*, *Cunninghamella elegans*, *Eurotium amstelodami*, *Mucor polymorphosporus*, *Penicillium simplicissimum*, *Streptomyces griseus* (Zhan et al. 2015; Gaur et al. 2014; Goswami et al. 2010; Liu et al. 2006; Parshikov et al. 2004, 2006; Zhan et al. 2002). Here, we first report the microbial transformation of artemisinin by *A. terreus*.

## Conclusions

In this work, we investigated the microbial transformation of artemisinin by *A. terreus*, and obtained two products, 1-deoxyartemisinin and 4α-hydroxy-1-deoxyartemisinin. This is the first report of microbial transformation of artemisinin by *A. terreus*.
